# Media Use of Young Children: The Perceptions of Public Health Nurses Working in Child Health Clinics

**DOI:** 10.1111/phn.13501

**Published:** 2024-12-02

**Authors:** Siiri Utriainen, Katri Vehviläinen‐Julkunen, Reeta Lamminpää

**Affiliations:** ^1^ University of Eastern Finland Kuopio Finland

**Keywords:** child health clinics, media use, parents, public health nurses, young children

## Abstract

**Objective:**

To describe the perceptions of public health nurses (PHNs) working in child health clinics on media use of young children and to investigate what are the perceptions of PHNs on adults’ role in young children's media use.

**Design:**

An electronic cross‐sectional survey with Likert‐scaled and open‐ended questions.

**Sample:**

The total of 183 PHNs working in Finnish child health clinics.

**Measurements:**

Statistical descriptive analysis for Likert‐scaled questions and thematic analysis for open‐ended questions were used.

**Results:**

Most (96.7%) PHNs agreed that media use was very common among young children. According to PHNs, the negative impacts of media use of young children included social, physical, and psychological impacts. The positive impacts included learning, communication, and access to information. PHNs stated that parents were most responsible for intervening in media use of young children, but PHNs have an important role in counseling families on it. Most PHNs (80.9%) stated that parents did not control media use of their children effectively.

**Conclusions:**

PHNs are aware of impacts of media use and its prevalence among young children. They agree to have an important role in counseling families in it. More work in research is needed to improve PHNs’ expertise and resources for them to be able to guide families in young children's media use. A wider perspective from other health care professionals working with families should be studied to constitute multi‐professional understanding.

## 1 Background

Technology development and digitalization have massively advanced in our society. Media, including televisions, computers, and the “new media,” such as smartphones, tablets, applications, and especially social media, are important parts of children's lives today. In recent years, media use has increased significantly and even children under the age of 3 have been using it. It takes up more time than studying, and even more time than sleeping (Badri et al. 2017; Coyne et al. 2017; Chong, Teo, and Shorey 2023).

Because of the increased screen time, The World Health Organization (WHO) recommends avoiding the screen time for children under the age of 2 and limiting the screen time of children aged 2–5 to 1 h or less. In Finland, recommendations for media use have been made by Finnish Institute for Health and Welfare in line with WHO suggesting children under the age of 2 to avoid using media at all, and children aged 2–4 to use media for not more than 1 h per day (Finnish Institute for Health and Welfare 2019). However, screen time of young children often exceed the limit stated by recommendations (Bahadur et al. 2021). Even 10% of 1‐year‐old children are regularly using digital media—mostly TV, but also the “new media” such as smart phones. Studies show that family screen time often predicts infant screen time (Durham et al. 2021).

Media use has both advantages and disadvantages for children. It is an essential part of learning and studying, it might be relaxing, and it is a huge part in children's social interactions (Limtrakul et al. 2018). It teaches children about relationships and empathy skills, as well as tolerance and understanding of other cultures (Richards, Caldwell, and Go 2015). However, media use also has multiple negative impacts on children. It includes plenty of harmful content such as violence, sex, and drug use. Media use might lead to problematic behavior, bullying, social exclusion, and health risks such as depression, exhaustion, obesity, and problems with concentration and sleep (Limtrakul et al. 2018; Richards, Caldwell, and Go 2015). High screen time among young children is also significantly associated with lower percentile ranks in cognition, language, and social‐emotional skills (Schwarzer et al. 2022).

According to Geurts et al. (2022), parents have a self‐interest in the media use of their children: they allow children to use media so that they can do other tasks without being interrupted by children, including having me‐time, managing children's behavior, avoiding discussions, using media themselves, and spending quality time with the partner (Geurts et al. 2022). Parents have an essential role in controlling young children's media use. However, the perspectives of parents regarding the screen time of children have not been studied enough (Chong, Teo, and Shorey 2023).

In Finland, parents’ expectations regarding media use among young children have been studied by interviewing the parents (*n* = 20). The results showed that parents expect more conversations, advice, and information regarding children's media use at child health clinics and parent's evenings (Moursad et al. 2023).

Public health nurses (PHNs), especially those working in child health clinics, have a significant role in guiding parents of young children to promote health. This includes talking with parents and families about media awareness and management (Schwarzer et al. 2022; Strasburger and Hogan 2018). Yet, there is a research gap in the perceptions of PHNs working in child health clinics regarding young children's media use (Coyne et al. 2017; Strasburger and Hogan 2018). Therefore, it is important to investigate what their perceptions of the topic are.

The purpose of this study is to describe the perceptions of PHNs working in child health clinics on media use of young children. Furthermore, the purpose is to investigate what are the perceptions of PHNs on adults’ role in young children's media use.

### 1.1 Child Health Clinics in Finland

Nearly all Finnish expectant mothers and families with under‐school‐age children use public health care services. These child health clinics in Finland are called Neuvolas and are organized by Finnish municipalities. Neuvola services include pregnancy and post‐pregnancy monitoring and health promotion; parental guidance; growth, development, and well‐being monitoring for under‐school‐age children; family support and well‐being promotion; and identification and family support for young children with potential special needs. Guidelines and recommendations are provided for child health clinic professionals to support their work with families by the Ministry of Social Affairs and Health. Currently, it is unclear if the guidelines include a mention of media use counseling (Ministry of Social Affairs and Health s.a.).

Children under school age have an opportunity to meet their child health clinic doctors and PHNs 15 times in the age of 4–6 weeks, 4 months, 8 months, 18 months, and 4 years. Three of these visits are general health check‐ups where the development, health, and well‐being of the child are assessed and the well‐being and needs of the family are evaluated, more commonly by PHNs. However, these check‐ups are multi‐professional, including evaluation from early childhood educational professionals on the well‐being and development of the child. All visits in Finnish child health clinics include health guidance. It is an essential duty of the PHNs to support the development of children, parenting, and parent‐child communication, as well as the mental health, relationships, and psychosocial well‐being of the parents. It also aims to promote social support networks for families (Finnish Institute for Health and Welfare s.a.).

### 1.2 Research Questions

In this study, young children are defined as children under school age (aged 0–6) in Finland. Media use refers to the use of smart devices and screen time including use of social media, TV, applications, and games.

The purpose of this study is to describe the perceptions of PHNs working in Finnish child health clinics on media use of young children. Furthermore, the purpose is to investigate what are the perceptions of PHNs on adults’ role in young children's media use.

The research questions are as follows:

1. What are the perceptions of PHNs on media use of young children?
2. What are the perceptions of PHNs on adults’ role in young children's media use?

## 2 Methods

This study was conducted using an electronic cross‐sectional survey which was then analyzed via both qualitative and quantitative methods. The qualitative part was implemented using thematic analysis and the quantitative part using statistical descriptive analysis. Both methods will be introduced in the analytic strategy section.

### 2.1 Design

Data were collected between spring 2019 and spring 2020 by convenience sampling in a cross‐sectional descriptive design. The Finnish Association of Public Health Nurses granted permission to the study in summer 2019.

### 2.2 Sample

Participation in the study was fully voluntary. The consent to participate in the study was implied, as by responding to the survey, participants consented to be part of the research. The participants were Finnish PHNs. They were recruited via Finnish Association of Public Health Nurses which includes over 7000 professionals. The contact person from the association sent the questionnaire to all members who were, according to their database, working as PHNs in child health clinics. The questionnaire was sent out three times to include enough participants: in April 2019, September 2019, and April 2020. In April 2019, the survey was sent to 955 PHNs.

### 2.3 Measures

The questionnaire included 30 questions; 10 questions about the background of participants, for example, age, working experience, and their own media use; 12 questions about the perception on media use among young children, including the benefits and disadvantages of media use, screen time and the role of parents; and 8 questions about the roles of PHNs in child health clinics in guiding children and families in media use. In these questions, there were 15 questions with five‐point Likert scale and five open‐ended questions. The open questions were about the advantages and disadvantages of media use among young children, the possible consequences due to increased screen time, the responsibilities of adults in controlling children's media use, and the development in the role of PHNs in child health clinics to improve the guidance on media use.

The questionnaire was designed based on earlier research (e.g., Domoff et al. 2019). The questionnaire was pre‐tested in spring 2019 with 10 PHNs via the Faculty of Health Science in University of Eastern Finland. The PHNs shared their opinions and proposals to improve the survey. These pre‐tested answers were not included in the data due to slight modifications in the survey after the pre‐testing.

### 2.4 Analytic Strategy

The data were analyzed with both quantitative and qualitative methods depending on the nature of the question. The Likert scale questions (*n* = 25), including questions about the background of PHNs (*n* = 10), were analyzed using statistical analysis (IBM SPSS statistics 27) focusing on the descriptive statistics (e.g., George and Mallery 2021), mostly on the frequencies and percentages. In the data analysis, some responses in the Likert scale were combined in the result section, that is, answers of “strongly agree” and “somewhat agree” were often combined as “strongly or somewhat agree.”

The open‐ended questions (*n* = 5) were analyzed using inductive thematic analysis (Braun and Clarke 2020). Participants’ answers were question by question, coded into simple phrases, which were then grouped into sub‐sub‐themes, sub‐themes, and in the end, main themes. These two main themes then formed the result section of this paper. Even though the analysis was inductive, the main themes were built so that their connection to the original data was preserved. The coding was made by using markings with different colors.

## 3 Results

### 3.1 Characteristics of Participants

A total of 183 PHNs participated in the survey. Despite the questionnaire was only distributed to those working in child health clinics, there were few (*n* = 11, 6%) that at the time of data collection were working in schools. This might have been the case if those participants had worked in child health clinics before but had changed their working place to schools. The response rate was 19.16%. All of the participants were females. The mean age of the participants was 42.9 years, and their average working experience was 12.7 years. The distribution of age, year of graduation, and working experience are presented in Table 1.

**TABLE 1:** Age, year of graduation, and working experience of PHNs (*n* = 183).

Age	*n*	%
<30	29	15.8
30–39	47	25.7
40–49	44	24.0
50–60	29	15.8
60 or older	13	7.1
Not specified	21	11.5

Year of graduation	n	%
1989 or earlier	16	8.7
1990–1999	37	20.2
2000–2009	49	26.8
2010–2019	78	42.6
Not specified	3	1.6

Working experience	*n*	%
5 or less	49	26.8
6–10	43	23.5
11–20	46	25.1
21–30	26	14.2
31–40	11	6.0
Not specified	8	4.4

Most PHNs worked in child health clinics (*n* = 155, 84.7%). The rest of the participants worked at schools (*n* = 11, 6.0%), both at schools and in child health clinics (*n* = 15, 8.2%), or in somewhere else (*n* = 1, 0.5%). Most PHNs worked as an employee (*n* = 163, 89.1%), and the rest of them as experts (*n* = 11, 6.0%), employees and managers (*n* = 3, 1.6%), employees and experts (*n* = 1, 0.5%), or other (*n* = 2, 1.1%).

A total of 139 PHNs (76.0%) had families with children under school age as their clients. The rest of them had children of all ages, or only school‐aged children, as clients. Over one‐third of the participants stated that they had approximately 200–299 clients per year (*n* = 70, 38.3%). Only 12 (6.6%) had less than 199 clients per year and 50 (27.3%) had 300–500 or more clients per year.

Most of PHNs (*n* = 125, 68.3%) stated that they spent time on media 1–2 h per day. Only 11 (6.0%) had screen time of 3 h or more, and 28 (15.3%) had less than an hour. Regardless of the age of respondents, the amount of time they spent daily on media did not vary much. The most popular medias among PHNs were WhatsApp (*n* = 181, 98.9%), Facebook (*n* = 151, 82.5%), and Instagram (*n* = 97, 53.0%). Snapchat (*n* = 23, 12.6%) and Twitter (*n* = 17, 9.3%) were forms of media less frequently used.

At the data analysis, two main themes were formed: (1) *the impacts of media use on young children*, with two subthemes: a) negative impacts of media use among young children, with three sub‐sub‐themes: social, physical, and psychological impacts; and b) positive impacts of media use among young children with three sub‐sub themes: learning, communication, and access to information; and (2) *the role of adults in controlling media use of children*, including the role of parents and PHNs. Examples of PHNs’ answers to open‐ended questions are given as direct quotes.

### 3.2 The Impacts of Media Use Among Young Children

A total of 96.7% PHNs (*n* = 177) agreed that media has been very visible in children's lives nowadays (with 67.8% strongly agreed and 28.4% somewhat agreed). Almost half of the participants (*n* = 85, 46.4%) somewhat agreed that there were more negative than positive impacts in media use among children. However, 25.1% (*n* = 46) stated that they somewhat disagreed with the claim, 24 (59%) of those who disagreed were under the age of 40. Almost all participants somewhat or strongly agreed (*n* = 166, 90.7%) that children under school age showed symptoms concerning excessive media use. Almost all participants agreed that young children were having too much screen time (with 23.0% strongly agreed and 54.6% somewhat agreed).

The disadvantages of media use among young children stated by PHNs varied a lot and were categorized into *social, physical, and psychological impacts*. Furthermore, the excessive screen time was often mentioned and related to all three themes.

According to the PHNs, there were several negative *social impacts* due to media use, including poor social skills, poor relationships and interaction with other people, increased bullying and inequality, increased social exclusion and loneliness, and pressure gained especially in social media. Grooming was also mentioned frequently. Negative *physical impacts* were also identified, including the decrease of playing and exercising, problems with eyesight, headaches, posture, and overweight, tiredness and sleep problems. Children becoming more passive due to media use resulting in decrease in physical activity was also mentioned frequently. Many PHNs also mentioned that children's withdrawal symptoms were common in the physical impacts of media use. Moreover, many young children were identified with negative *psychological impacts* related to the exposure of inappropriate material and content in social media and excessive screen time. These psychological impacts included addiction, restlessness and anxiety, problematic behavior such as aggression, concentration difficulties, mental issues (e.g., depression), frustration, hyperactivity, learning disabilities, and self‐esteem issues. PHNs also stated that due to media use, the development of language and reading skills became slower, imagination became poorer, and the tolerance of boredom decreased among children.

“A child might see the kind of content that is not meant to be for him/her” PHN, age 34, 10 years of working experience

“Excessive media use steals time from other important activities, such as outdoor activity” PHN, 2 years of working experience

However, few *positive impacts* of media use related to learning and communication among young children were also identified. Positive impacts on *learning* included the development of language and reading skills, the evolution of the use of digital devices and information retrieval, and better problem solving, coordination and prestidigitation skills. PHNs also stated that social media and smart devices have included many pedagogical and educational applications, stories, and games. Positive impacts were also identified in *communications*. Social media helped children to keep contact with their peers and other relations, connected children and parents by playing games together, and has been a great platform for making contacts with new people.

“There is a lot of good, educational information in media” PHN, age 41, 15 years of working experience

“Many games are supportive when practicing reading and writing” PHN, age 31, 3 years of working experience

### 3.3 The Role of Adults in Controlling media Use of Children

Based on the results of the survey, two themes were found concerning the role of adults in controlling media use among young children and were mostly formed based on responses to the Likert‐scale questions and two open questions. These answers were then coded into to sub‐themes; *the role of parents and PHNs*.

Regarding *parental role*, most participants strongly or somewhat agreed that parents did not control media use of their children effectively (*n* = 148, 80.9%), while participants who had worked as PHNs for less than 10 years, more often disagreed (*n* = 16, 35.2%). More than two‐thirds of PHNs strongly or somewhat agreed that parents did not know how to intervene media use of children (*n* = 125, 68.3%). However, a total of 39 (21.3%) somewhat disagreed with this claim. Most participants also agreed that parents needed guidance from PHNs to control media use of young children (29.5% [*n* = 54] strongly agreed and 55.7% [n = 102] somewhat agreed). Also, for those who disagreed that PHNs were most responsible for intervening in children's media use, a total of 36 out of 50 (72%) PHNs stated that the primary responsibility belonged to parents or caregivers. The rest 14 (28%) stated that the responsibility belongs to professionals such as PHNs, doctors, early childhood education professionals, and others working with families.

“It's parents’ responsibility, but they need attention, information, and guidance” PHN, age 42, 7 years of working experience

“The full responsibility belongs to parents. In child health clinics it [media use] can be brought up and guided” PHN, age 31, 9 years of working experience

Regarding the perceptions of participants concerning *PHNs’ roles*, expertise, and resources in guiding parents in child health clinics on media use among young children, it was clearly an essential part of the duty of PHNs to guide and intervene media use among young children. Majority of PHNs (*n* = 131, 71.6%) somewhat or strongly agreed that guiding parents in this topic belonged especially to PHN duties in child health clinics and 167 (91.3%) somewhat or fully agreed that it was the responsibility of PHNs to intervene excessive media use of children. A total of 148 (80.9%) participants agreed that guiding families in media use of young children was already an essential part of their duties. Even if it was or should have been their responsibilities, the resources such as time, expertise, education, information, and material, were clearly inadequate. Most participants thought that PHNs in child health clinics did not have enough time to guide families in media use of children. A total of 151 (82.5%) somewhat or strongly disagreed that there is enough time for that. A total of 117 (64.0%) somewhat or strongly disagreed that PHNs in child health clinics had enough expertise for media use guidance. Also, the need for education and information for guiding families in media use was identified: a total of 123 (67.2%) somewhat or strongly disagreed that there was enough education and information. A total of 100 (54.6%) somewhat or strongly disagreed that there was enough material for guiding parents in media use of young children. The responses in Likert‐scale questions strongly correlated with that of the open questions on how the work in child health clinics should be developed to enhance parental guidance in media use of young children. PHNs stated that they need more training and information, and resources such as time and material to support their work. Many participants also stated that guiding families in media use of young children should be emphasized more as part of the duties of PHNs in child health clinics.

“More resources and education for public health nurses are needed” PHN, age 26, less than 1 year of working experience

“Written material and national recommendations are needed” PHN, 20 years of working experience

## 4 Discussion

The findings from this study show that PHNs in Finnish child health clinics have nuanced understanding of the media's role in the lives of young children. PHNs recognize both the potential benefits and the risks associated with young children's media use, and the complexity of PHNs’ role in guiding families through these challenges.

The majority of PHNs agreed that media use is highly prevalent among young children, reflecting a broad awareness of its prevalence in modern childhood. The reported negative impacts of young children's media use include social, physical, and psychological challenges. The concern about excessive screen time leading to reduced physical activity and health issues is consistent with existing literature; the prolonged media use can affect physical health, social skills, and emotional well‐being (Strasburger, Jordan, and Donnerstein 2010). Despite recognizing these negative impacts, PHNs also acknowledge some positive aspects of media use among young children, such as enhanced learning and communication opportunities. Moderation and context play critical roles in determining the overall impact of media use (Chong, Teo, and Shorey 2023).

The study reveals a great concern among PHNs regarding the effectiveness of parental control over media use of young children. The results showed that most PHNs believe parents are not adequately managing their children's media use. Therefore, there seems to be a gap in parental strategies or resources. Previous studies also indicate that parents are struggling with setting and enforcing media use boundaries for their children (Gentile et al. 2009; Chong, Teo, and Shorey 2023).

PHNs feel a responsibility to guide parents in managing young children's media use. Yet they also report feeling under‐resourced and inadequately trained for this it. This result highlights a need for enhanced support for PHNs, including more training, time, and resources to effectively counsel families. Guiding media use in child health clinics should be a more prominent part of PHNs’ role reflecting a broader recognition of the importance of this issue in child development and health (Schwarzer et al. 2022). The identified gaps in expertise, time, and resources suggest that while PHNs are aware of their role in media guidance, systemic support is lacking. The reported inadequacies in training and educational materials point to a need for better support systems and updated guidelines to assist PHNs in their counseling role. More resources and structured educational materials are needed for a more robust framework to support PHNs (Strasburger and Hogan 2018).

PHNs working in public health services, especially in child health clinics, should raise media awareness and management both in parents and children (Schwarzer et al. 2022; Strasburger and Hogan 2018). PHNs in child health clinics should receive more training about children's media use to acquire transferable skills to guide parents to control it. (Badri et al. 2017; Strasburger and Hogan 2018).

Moreover, the need for a multi‐professional approach is critical. Integrating perspectives from other healthcare professionals and creating a strategy for managing media use of young children across different domains of child health could enhance the effectiveness of guidance provided to families. Thus, future research should explore how other healthcare professionals, such as pediatricians and early childhood educators, find the issue of media use and collaboration with PHNs to form a comprehensive and multi‐disciplinary work. Additionally, studies investigating effective training programs and resources for PHNs could provide valuable insights to how to equip them for this crucial aspect of young children's health (Bahadur et al. 2021; Chong, Teo, and Shorey 2023).

While Finnish PHNs are clearly aware of the significant role media plays in young children's lives, there is a clear need for increased support and resources to enable them to fulfill their advisory roles effectively. Addressing these gaps could enhance the overall efficacy of media use guidance provided to families, ultimately supporting better developmental outcomes for young children (Moursad et al. 2023).

### 4.1 Limitations of the Study

This study has a few limitations. The questionnaire was developed for this study and was pre‐tested once before sending out to participants. The response rate of this study was 19.16% and the survey was sent out to participants three times during 2019–2020. There might be a few explanations for the low response rate. In spring 2020, the first wave of COVID‐19 pandemic hit Finland, and many restrictions were set to reduce the spreading of an infection. The restrictions also affected the service of child health clinics.  According to Wu, Zhao, and Fils‐Aime (2022), having a reasonable number of participants might be more important for the study than a high response rate; in this study, there were a good number of respondents (*n* = 183). One reason may be that participants were contacted through The Finnish Association of Public Health Nurses which includes over 7000 professionals, and the list might not be up to date. As the questionnaire was sent out to participants in 2019 and 2020, the Finnish child health clinics might have developed further since then. However, there is no data available on the change or increase of media use counseling after 2020.

## 5 Conclusions

This study highlights the significant role that PHNs have in addressing the effects of media use among young children. The findings reveal that PHNs are aware of the media's presence in children's lives and its impacts—both positive and negative. While acknowledging the benefits such as enhanced learning and communication, PHNs also recognize the considerable risks, including social, physical, and psychological challenges of excessive media use.

Despite their awareness, PHNs reported facing challenges related to inadequate resources, time and training, which challenges their ability to effectively guide families in media use. There is a need for enhanced support and resources to empower PHNs in their role in guiding families. Additionally, the study highlights the necessity of a multi‐professional approach.

Future efforts should focus on developing more training programs and resources for PHNs regarding young children's media use, as well as fostering collaboration with other healthcare professionals. By addressing these gaps, we can better support families in managing media use of young children and promoting healthier developmental environments for them.

## Ethics Statement

The authors have nothing to report

## Conflicts of Interest

The authors declare no conflicts of interest.

## Data Availability

Research data are not shared.
